# Testing a Microarray to Detect and Monitor Toxic Microalgae in Arcachon Bay in France 

**DOI:** 10.3390/microarrays2010001

**Published:** 2013-03-05

**Authors:** Jessica U. Kegel, Yolanda Del Amo, Laurence Costes, Linda K. Medlin

**Affiliations:** 1 Marine Biological Association of the United Kingdom, The Laboratory, Citadel Hill, Plymouth, PL1 2PB, UK; 2 Université Bordeaux 1, UMR CNRS EPOC 5805, Station Marine d’Arcachon, 2 rue du Prof. Jolyet F 33120 Arcachon, France; E-Mails: y.delamo@epoc.u-bordeaux1.fr (Y.D.A.); l.costes@epoc.u-bordeaux1.fr (L.C.)

**Keywords:** oligonucleotide microarrays, molecular monitoring, harmful algal blooms, HABs, toxic microalgae, 18S/28S ribosomal RNA, LSU/SSU, RNA hybridization, environmental water samples

## Abstract

Harmful algal blooms (HABs) occur worldwide, causing health problems and economic damages to fisheries and tourism. Monitoring agencies are therefore essential, yet monitoring is based only on time-consuming light microscopy, a level at which a correct identification can be limited by insufficient morphological characters. The project MIDTAL (Microarray Detection of Toxic Algae)—an FP7-funded EU project—used rRNA genes (SSU and LSU) as a target on microarrays to identify toxic species. Furthermore, toxins were detected with a newly developed multiplex optical Surface Plasmon Resonance biosensor (Multi SPR) and compared with an enzyme-linked immunosorbent assay (ELISA). In this study, we demonstrate the latest generation of MIDTAL microarrays (version 3) and show the correlation between cell counts, detected toxin and microarray signals from field samples taken in Arcachon Bay in France in 2011. The MIDTAL microarray always detected more potentially toxic species than those detected by microscopic counts. The toxin detection was even more sensitive than both methods. Because of the universal nature of both toxin and species microarrays, they can be used to detect invasive species. Nevertheless, the MIDTAL microarray is not completely universal: first, because not all toxic species are on the chip, and second, because invasive species, such as *Ostreopsis*, already influence European coasts.

## 1. Introduction

Worldwide every year, fisheries, aquaculture, human health and tourism are threatened by harmful algal blooms (HABs) in marine, brackish, as well as continental waters. Although most phytoplankton species are benign, about 2% of them can cause harm through the production of toxins or by an excessive accumulated biomass, which can affect co-occurring organisms and alter food-web dynamics [1,2]. In addition to the ecological and economic damages, public health is also at risk: the consumption of shellfish that have fed on toxic phytoplankton and accumulated toxins, and exposure to the aerosols of HAB toxins can cause illness or even mortality. Depending on the species, it can take only a few toxic cells per liter to poison shellfish and make them unsuitable for human consumption [3]. Monitoring of microalgae is therefore required by all countries with a marine coastline. HAB monitoring programs are currently based on cells identified and counted by light microscopy and on the mouse bioassay for detecting biotoxins. The mouse bioassay for the detection of phytoplankton toxins in shellfish has recently been banned by the European Commission (July 2011), and there is a mandatory replacement by chemical methods (Liquid Chromatography-Mass Spectrometry, LC-MS) in the next three years.

However, the effectiveness of monitoring programs using light microscopic identification is limited by the fact that it is time consuming and that morphology, as determined by light microscopy, may be insufficient to give definitive species and toxin attribution. Thus, there is a need to implement molecular methods to ensure a fast and reliable species identification. Monitoring for toxic species using molecular techniques advances the state of knowledge for detection of harmful species because more samples can be analyzed in a shorter time period and with greater accuracy. Within the actual context of the dramatic decreasing number of taxonomic experts of phytoplankton [4], which are, notwithstanding, essential for other ecological studies, such techniques offer important advances. This is of particular interest for potentially toxic algae, because the difficulty in determining their exact identification by using light microscopy can have disastrous consequences for human health. Microarrays offer a near real-time ecosystem analysis and offer broader ecological interpretation of how key species, such as toxic algae, can extend their geographical distribution with climate change or can become invasive after introduction from remote areas [5]. Microarrays offer the most expeditious method to have high sample throughput with highly accurate species detection, in a universal approach [6,7,8]. In the FP7 EU project, MIDTAL (Microarrays for the Detection of Toxic Algae), an earlier protocol for detection of toxigenic microalgae by Gescher *et al.* [9] was optimized. Microarrays (as phylochips) detect multiple species simultaneously using species-specific probes that have been applied primarily for the detection of bacteria [10,11,12,13]. At present, 140 probes for various toxic algal species at various taxonomic levels are spotted onto the current generation of the MIDTAL microarray. As part of the MIDTAL project, the primary goal was to be able to infer cell numbers from the molecular signal to provide an early warning system for toxic algae. Because the MIDTAL microarray is a universal array that can be used globally, it offers a real possibility of detecting invasive species, especially in view of global warming where warm temperate species are moving northward, e.g., *Gamberiodiscus*.

**Figure 1:**
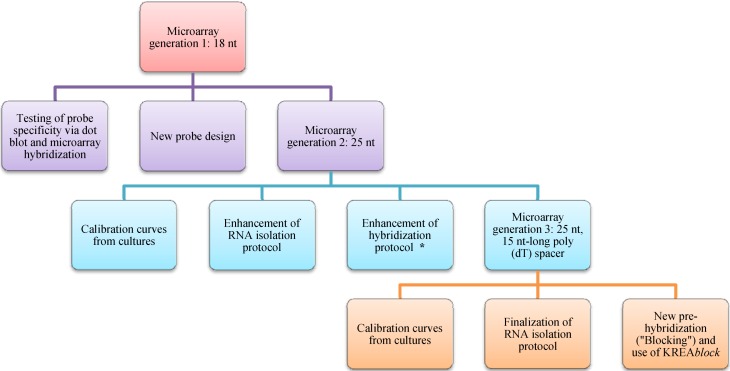
Scheme of the development of the MIDTAL microarray. The scheme pictures the different microarray generations with its different probes, tests and enhancements of protocols (RNA and hybridization). (* Higher temperature during 3rd washing step).

In this study, we show the effectiveness of using microarrays for the detection of toxic algae and its combination with toxin detection. We compare these results with light microscopy data from a regular French monitoring network of toxic phytoplankton. The microarray used in this study represents the third generation array developed within the EU-MIDTAL project. In generation one, probes (18–22 nt) developed for Fluorescent *in situ* Hybridization (FISH) were used directly; in generation two, these FISH probes, and any newly designed probes, were lengthened to 25 or more nt; in generation three, an additional poly-T spacer to lift the probes farther above the surface was tested and optimized (Figure 1). At each generation, minor changes in the hybridization protocol were made and a final optimized protocol can be found in Lewis *et al.* [14].

## 2. Experimental Section

### 2.1. Field Sampling

**Figure 2:**
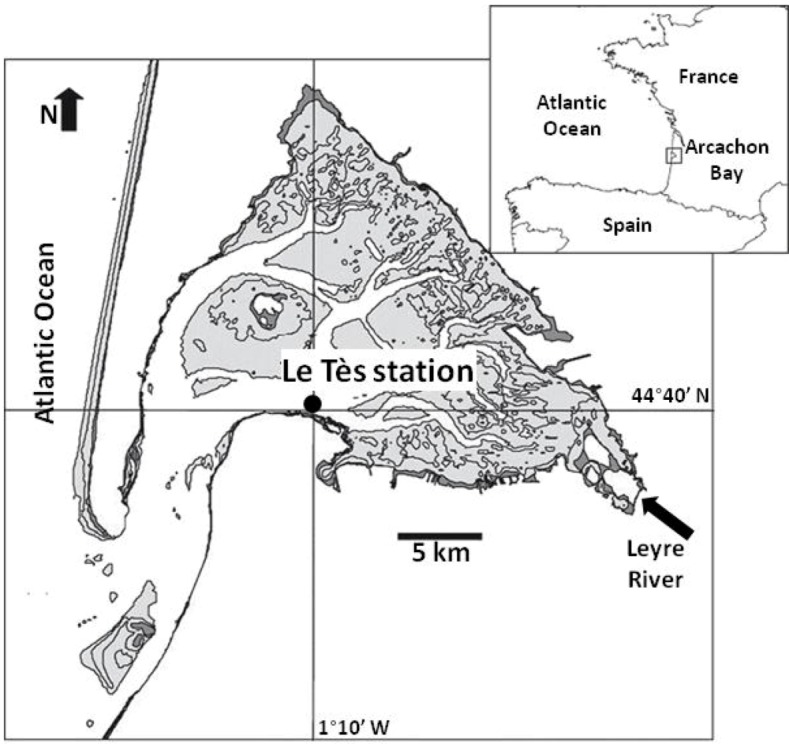
Sampling sites in Arcachon Bay (France): the station Tès (Teychan).

In 2011, water samples from the sub-surface (1 m depth) were collected at Arcachon Bay in France (Figure 2) between July and October for microarray analysis (Table 1). The sampling site termed Tès (1°10'00 W, 44°40'00 N), is directly located in front of the town of Arcachon inside the bay. Data of toxic, harmful, and other phytoplankton abundances is provided by IFREMER (Ifremer/Quadrige^2^/Rephy DATA) from the paired station named Teychan (1.5 km from Tès). Cell counts were done as previously described by Medlin and Schmidt [15] and Kegel *et al*. [16]. 

**Table 1:** Information about field samples taken at Arcachon Bay like sample name, sample date, filtered volume, total extracted RNA and degree of labeling (DoL).

Sample name	Sample date	Volume filtered (L)	Total RNA extracted (µg)	DoL
1A	24/07/2011	3.3	7.46	2.2
2A	08/08/2011	3.0	9.48	2.0
3A	22/08/2011	3.3	9.52	1.9
4A	04/10/2011	3.25	10.66	2.2
6A	20/10/2011	3.3	13.82	2.2

For the microarray analysis, a minimum of three liters (Table 1) were filtered onto 3 µm nitrocellulose filters (47 mm) in triplicate. For each sampling date, the first and second replicated filter was transferred into cryogenic vials containing 1 mL of TRI Reagent (Sigma-Aldrich). Those samples were snap frozen and stored at –80 °C until further process for RNA extraction. Toxicity was measured by one of the Partners (Queens University Belfast, UK) with a newly developed multiplex optical Surface Plasmon Resonance biosensor (Multi SPR) in parallel with the enzyme-linked immunosorbent assay (ELISA) [17]. The target toxins are domoic acid (DA) for amnesic shellfish poisoning (ASP), okadaic acid (OA) and dinophysistoxins (DTXs) for diarrhetic shellfish poisoning (DSP) and saxitoxin (STX) for paralytic shellfish poisoning (PSP) toxin analogs. Therefore, the third replicated filter was transferred into cryogenic vials without TRI Reagent and sent frozen to Queens University Belfast who was responsible for the toxin measurements.

### 2.2. RNA Extraction

RNA extraction was done with minor changes to that presented in Kegel *et al.* [16]. Briefly, acid-washed glass beads (300 µm) and 500,000 cells of *Dunaliella tertiolecta*-strain UIO226 (stored in TRI Reagent) as a control were added to the samples and the samples were bead-beaten twice for 1 min at 4,800 oscillations/min (BioSpec Mini Bead Beater). Cell-TRI Reagent mixture was transferred into a new microcentrifuge tube, vortexed for 15 s and left to stand at room temperature (RT) for 10 min. After another 15 s of vortexing, samples were incubated at 60 °C for 10 min in a Thermoshaker at maximum speed. Samples were vortexed again for 15 s and then transferred into pre-spun Phase Lock Gel Heavy 2 mL tubes (5 Prime; 12,000 g for 30 s). After the addition of 100 µL of 1-bromo-3-chloropropane (BCP) to the samples, the tubes were shaken thoroughly for 15 s. Samples were incubated at RT for 5 min and centrifuged (12,000 ×*g*) for 15 min at 4 °C. The upper phase was mixed gently with 200 µL chloroform and centrifuged (12,000 ×*g*) for 2 min at 4 °C. The aqueous phase was then transferred to a fresh 2 mL RNase-free tube. Equal volumes of isopropanol were added, vortexed for 15 s and incubated for one hour at −20 °C. After incubation, samples were centrifuged (12,000 ×*g*) for 15 min at 4 °C. Supernatant was quickly removed and pellets were washed three times with 1 mL ethanol (75%): ethanol was added, vortexed for 5 s, centrifuged (12,000 ×*g*) for 10 min at 4 °C, and supernatant carefully removed. Following the third wash, the supernatant was completely removed and the pellet was air-dried for 5 min. The pellet was dissolved in 100 µL of RNase-free water. To get rid of TRI Reagent residuals, samples were precipitated with 0.5 volume of 7.5 M NH_4_Ac and 2 volumes of ice-cold ethanol (absolute, stored at −20 °C). The mixture was vortexed and incubated at −80 °C for 1.5 h. Immediately after incubation, samples were centrifuged at 4 °C and max. speed for 20 min. The supernatant was removed; the pellet was washed in 500 µL of 70% ice-cold ethanol (stored at −20 °C) and centrifuged for 5 min at max. speed. The washing step was repeated and the pellet was air-dried for 30–60 min. The RNA was re-suspended in 50 µL nuclease-free water and its concentration and integrity was measured by NanoVue spectrophotometer (GE Healthcare) and Agilent Bioanalyzer 2100 (Agilent Biotechnologies). Samples were snap-frozen in liquid nitrogen and stored at −80 °C until further use.

### 2.3. RNA Labeling and Fragmentation

The PlatinumBright Infrared Labeling Kit from KREATECH (Amsterdam, Netherlands) was used to label 1.5 µg RNA of field sample using 2 µL ULS dye and 2 µL 10× labeling solution in a total volume of 20 µL. Samples were labeled by incubation for 30 min at 85 °C. After incubation, samples were placed on ice and spun down and then purified with KREA*pure* columns (KREATECH) according to the manufacturer’s instructions. Concentration and incorporation of the dye was measured by a NanoVue (GE Healthcare). The DoL (degree of labeling) was calculated and was between 1.9 and 2.2% (Table 1). RNA was fragmented by adding 1/10 volume of RNA fragmentation buffer (100 mM ZnCl_2_ in 100 mM Tris-HCL, pH 7.0) and an incubation of 15 min at 70 °C. The reaction was stopped with the addition of 1/10 volume of 0.5 M EDTA (pH 8.0) and the samples were placed on ice. The RNA was fragmented to reduce the effect of the secondary structure on the accessibility of the probe. Despite this fragmentation, we still have heterogeneous probe sensitivity, which reflects the influence of the secondary structure and we can only partially overcome this by fragmenting the RNA to remove the strongest secondary structure formations.

### 2.4. Microarray Design

Probe design was done with the open software package ARB [18]. All oligonucleotides including the positive and negative controls were synthesized by Thermo Fisher Scientific (Ulm, Germany) with a C6 aminolink at the 5' end of the molecule. The probes had a length between 18 and 25 nt and a 15 nT-long poly (dT) tail following the NH_2_ link at the 5' end. Table 2 shows a list of the probes and their targets. The complete hierarchy for each probe can be found in the GPR-Analyzer which is available online at http://folk.uio.no/edvardse/gpranalyzer. The probe sequences are patent pending and a commercial kit will soon be available from Kreatech containing the array and all reagents for hybridization. Epoxy-coated slides (Genetix or Schott) of MIDTAL version 3.2 were printed using a pin printer VersArray ChipWriter Pro (Bio-Rad Laboratories GmbH, Munich, Germany) and split pins (Point Technologies, Inc., CO) as described by Kegel *et al.* [16]. One array contained 136 different probes and 4–8 replicates, as well as three negative (NEGATIVE1_dT, NEGATIVE2_dT, NEGATIVE3_dT), one positive control (TBP = TATA-box binding protein), Poly-T-Cy5 (spotting control), and two internal controls (DunGS02_25_dT and DunGS05_25_dT for *Dunaliella tertiolecta*) (MIDTAL ver3.2). After spotting, slides were incubated for 30 min at 37 °C and then stored at −20 °C.

Printing of MIDTAL slides version 3.3 was done by Scienion AG using a sciFlexarrayer S11 and epoxy-coated slides from Genetix. One array contained eight replicates of 140 different probes including the seven controls stated above. After printing, the slides were transferred to a 75% humidity chamber, kept there overnight at RT, and stored afterwards in a sealed aluminum bag refilled with argon at 4 °C.

**Table 2:** Summary of probes designed or modified from published FISH probes and used to form the third generation of the MIDTAL microarray, including the targeted species, and whether it was made from the 18S or 28S rRNA gene. Probe sequences are not provided because the microarray is patent pending and will soon be commercially available from Kreatech, Amsterdam, The Netherlands. A complete taxonomic ordering of the probes can be seen in the GPR-Analyzer program and the MIDTAL hierarchy file that comes with that program.

Probe Name	Targeted Taxon	Gene
**Controls**		
DunGS02_25_dT	*Dunaliella* spp.	18S
DunGS05_25_dT	*Dunaliella* spp.	18S
**Higher group-level probes**		
EukS_328_25_dT	Eukaryotes	18S
EukS_1209_25_dT	Eukaryotes	18S
HeteroS01_25_dT	Heterokonta	18S
PrymS01_25_dT	Prymnesiophyta	18S
**Class-level probes**		
PrymS03_25_dT	Prymnesiophyceae	18S
DinoB_25_dT	Dinophyceae (incl. Apicomplexa)	18S
DinoE12_25_dT	Dinophyceae (incl. Apicomplexa)	18S
**Clade-level probes**		
DphyexacutaFS01_25_dT	Dinophysiaceae (*Dinophysis* + *Phalacroma*)	18S
DphyFS02_25_dT	Dinophysiaceae (*Dinophysis* + *Phalacroma*)	18S
PdeliD02_25_dT	*P. delicatissima* all clades	28S
Clade 01new_25_dT	*Prymnesium* B1 clade	18S
Clade01old_25_dT	*Prymnesium*	18S
ProroPKD01_25_dT	*Prorocentrum* planktonic clade	28S
ProroFPS01	*Prorocentrum* planktonic clade	18S
ProroFBS02_25_dT	*Prorocentrum* benthic clade	18S
ProroFBS01	*Prorocentrum* benthic clade	18S
**Genus-level probes**		
PsnGS01_25_dT	*Pseudo-nitzschia*	18S
PsnGS02_25_dT	*Pseudo-nitzschia* + *Fragilariopsis*	18S
PSN+FRAGS02-25new_dT	*Pseudo-nitzschia* + *Fragilariopsis*	18S
PSN no pungens_25_dT	*Pseudo-nitzschia* no pungens	18S
PSN + some Frags_25_dT	*Pseudo-nitzschia* + some *Fragilariopsis*	18S
KareGD01_25_dT	*Karenia*	28S
AlexGD01_25_dT	*Alexandrium*	28S
DphyGD01_25_dT	*Dinophysis* in part	28S
DphyGD02_25_dT	*Dinophysis*	28S
PschGS01_25_dT	*Pseudochattonella* (genus)	18S
PschGS04_25_dT	*Pseudochattonella* (genus)	18S
PschG05_25_dT	*Pseudochattonella* (genus)	18S
DphyGS01_25_dT	*Dinophysis* genus sensu stricto	18S
DphyGS02_25_dT	*Dinophysis* genus sensu stricto	18S
DphyGS03_25_dT	all *Dinophysis* and *Phalacroma*	18S
DphyGS04_25_dT	all *Dinophysis*	18s
KargeD01_25_dT	*Karlodinium* genus	28S
AzaGD01_dT	*Azadinium* genus	28S
AzaGD03_dT	*Azadinium* genus	28S
AzaGS01_dT	*Azadinium* genus	18S
AzaGS02_dT	*Azadinium* genus	18S
**Species-level probes**		
AtamaS01_25_dT	*Alexandrium* NA,WE,TA, species complex	18S
AminuS01_25_dT	*Alexandrium minutum*	18S
ATNA_D01_25_dT	*A. tamarense* (North America)	28S
ATNA_D02_25_dT	*A. tamarense* (North America)	28S
ATTA _D01_25_dT	*A. tamarense* (Temperate Asian)	28S
AostD01 _25_dT	*A. ostenfeldii*	28S
AostS02 _25_dT	*A. ostenfeldii*	18S
CpolyS01_25_dT	*Chrysochromulina polylepis*	18S
PparvD01_25_dT	*Prymnesium parvum*	28S
Prymparv01_25_dT	*Prymnesium parvum*	18S
KbreD03_25_dT	*Karenia mikimotoi* and *brevis*	28S
KbreD04_25_dT	*K. mikimotoi* and *brevis*	28S
KmikiD01_25_dT	*K. mikimotoi*	28S
KbreD05_25	*K. brevis*	28S
LSKbre0548A25_dT	*K. mikimotoi* and *brevis*	28S
KmGcS06_25_dT	*K. mikimotoi*, *Gymnodinium catenatum*, *cf. Chatonella sp.*	18S
KbreD03c_25_dT	Competitor *K. mikimotoi* and *brevis*	18S
KbreD04_25c_dT	Competitor *K. mikimotoi* and *brevis*	28S
SSKbre1448A25_dT	*K. brevis*	18S
SSKbre1448A25c_dT	*K. brevis*	18S
LSKBre0548A25c_dT	*K. brevis*	28S
SSGcat0826A27_dT	*Gymnodinium catenatum*	18S
LSGcat0270A24_dT	*G. catenatum*	28S
GcateS01_25_dT	*G. catenatum*	18S
KveneD01_25_dT	*Karlodinium veneficum*	28S
KveneD02_25_dT	*Karlodinium veneficum*	28S
KveneD03_25_dT	*Karlodinium veneficum*	28S
KveneD04_25_dT	*Karlodinium veneficum*	28S
KveneD05_25_dT	*Karlodinium veneficum*	28S
KveneD06_25_dT	*Karlodinium veneficum*	28
PlimaS01_25_dT	*Prorocentrum lima*	18S
PlimaFD01_2_dT5	*P. lima*	28S
PmicaD02_25_dT	*P. micans*	28S
PminiD01_25_dT	*P. minimum*	28S
PmacuS01	*P. maculosum* and *belizeanum*	18S
PmacuD01	*P. maculosum*	28S
PmacuD02	*P. maculosum*	28S
PrathD01	*P. rathymum* and *mexicanum*	28S
PrathD02	*P. rathymum* and *mexicanum*	28S
DacumiD02_25_dT	*Dinophysis acuminata*, *dens* and *sacculus*	28S
DacutaD02_25_dT	*Dinophysis acuta* and *fortii*	28S
DacumiS01_25_dT	*Dinophysis acuminata*	18S
DacutaS01_25_dT	*Dinophysis acuta*	18S
DnorvS01_25_dT	*Dinophysis norvegica*	18S
PausserD01_25_dT	*Pseudo-nitzschia australis* and *seriata*	28S
PmulausD01_25_dT	*P. australis* and *multistriata*	28S
PcaserausD02_25_dT	*P. australis, seriata, deli2*	28S
PcaserausD03_25_dT	*P. australis, seriata, calliantha*	28S
PfraucalD02_25_dT	*P. fraudulenta, subfraudulenta, calliantha*	28S
PcaciD01_25_dT	*P. caciantha*	28S
PcaciD02_25_dT	*P. caciantha*	28S
PcaciD04_25_dT	*P. caciantha*	28S
Pcal1D01_25_dT	*P. calliantha*	28S
PmanD01_25_dT	*P. manii*	28S
Pman2D02_25_dT	*P. manii*	28S
Pman2D03_25_dT	*P. manii*	28S
Pman2D05_25_dT	*P. manii*	28S
Pdel4D01_25_dT	*P. cf. delicatissima* Clade4	28S
Pdel4D02_25_dT	*P. cf. delicatissima* Clade4	28S
Pdel3B_25_dT	*P. delicatissima* clade 3 + *micropora*	28S
Pdel3A_25_dT	*P. delicatissima* clade 3 + *micropora*	28S
CompPdel3_25_dT	Competitor Pdel3A	28S
Pdel1D01_25_dT	*P. delicatissima* Clade1	28S
Pcaldel2D01_25_dT	*P. delicatissima* Clade2	28S
PcaldelD03_25_dT	*P. delicatissima* Clade2 and *calliantha*	28S
Pdel4D03_25_dT	*P. delicatissima* Clade4	28S
PgalaD01_25_dT	*P. galaxiae*	28S
PgalaD02_25_dT	*P. galaxiae*	28S
PgalaD04_25_dT	*P. galaxiae*	28S
PmultS01_25_dT	*P. multiseries*	18S
PmultD02_25_dT	*P. multiseries*	28S
PmultcalD01_25_dT	*P. multiseries* and *calliantha*	28S
PmultcalD03_25_dT	*P. multiseries* and *calliantha*	28S
PmultcalD04_25_dT	*P. multiseries* and *calliantha*	28S
PcalfrauD04_25_dT	*P. fraudulenta* and *multistriata*	28S
PmulaD03_25_dT	*P. multistriata*	28S
PmulacalD02_25_dT	*P. multistriata* and *calliantha*	28S
PpdeD01_25_dT	*P. pseudodelicatissima* and *cuspidata*	28S
PpdeD02_25_dT	*P. pseudodelicatissima* and *cuspidata*	28S
PpungcalS01_25_dT	*P. pungens* and *calliantha*	18S
PpungcalD02_25_dT	*P. pungens* and *calliantha*	28S
PpungcalD04_25_dT	*P. pungens* and *calliantha*	28S
PsercalD01_25_dT	*P. seriata* and *calliantha*	28S
CtoxS05_25_dT	*cf. Chatonella sp.*	18S
CtoxiS07_25_dT	*cf. Chatonella sp.*	18S
CtoxiS09_25_dT	*cf. Chatonella sp.*	18S
PfarD01_25_dT	*Pseudochattonella farcimen*	28S
PverD01_25_dT	*Pseudochattonella verruculosa*	28S
SSHaka0193A25_dT	*Heterosigma akashiwo*	18S
SSHaka0200A25_dT	*H. akashiwo*	18S
LSHaka0544A25b_dT	*H. akashiwo*	28S
LSHaka0268A25_dT	*H. akashiwo*	28S
LSHaka0544A25c_dT	*H. akashiwo*	28S
LSHaka0548A25_dT	*H. akashiwo*	28S
LSHaka0329A25_dT	*H. akashiwo*	28S
LSHaka0358A24_dT	*H. akashiwo*	28S

### 2.6. Microarray Hybridization

Before use, slides were blocked by incubating the DNA chips in a blocking solution (0.02% SDS, 2× SSC) for 20 min at 50 °C and ~70 rpm in the dark. The slides were washed once in ddH_2_O for 10 min at 50 °C and twice always in fresh ddH_2_O for 15 min at RT and ~70 rpm in the dark. The slides were dried by centrifugation in a glass dish for 3 min at 900 rpm and stored in the fridge (possible for up to two month).

Labeled field samples (1 µg RNA) were mixed with 30 µL of 2× hybridization buffer, 3 µL Poly-dA (1 µM), 10 ng TBP-control and adjusted with nuclease-free water to 45 µL. Poly-dA is added to block the poly-T spacer on the probe and TBP is the TATA box gene fragment added as the positive hybridization control. The labeled RNA was then denatured for 5 min at 94 °C. After denaturation, the samples were shortly placed on ice and 15 µL of KREA*block* (background blocker from KREATECH) was added. Slides were placed into an array holder; coverslips (LifterSlips, Erie Scientific, USA) were cleaned and placed onto the microarrays. Half of the hybridization mixture (30 µL) was added to one microarray. Hybridization was carried out for 1 h at 65 °C in a 50 mL Falcon tube containing a wet Whatman paper. The DNA chips were washed three times and shaken (~70 rpm) in the dark under stringent conditions. The washings were always undertaken for 10 min. The incubation in the first washing buffer (2× SSC/10 mM EDTA/0.05% SDS) and the second washing buffer (0.5× SSC/10 mM EDTA) was done at room temperature. The incubation in the third washing buffer (0.2× SSC/10 mM EDTA) was done at 50 °C.

### 2.7. Data Analysis

Obtained fluorescent signals and the surrounding background intensity were calculated by superimposing a grid of circles (midtal_ver32_20110429.gal or MIDTAL_V3.3.gal) onto the scanned image using the GenePix 6.0 software. First results were processed through the phylochip analyzer program to generate a hierarchy file to establish the hierarchical levels of the probes on the chip [19]. The hierarchy file and hybridization results were then progressed with the GPR-Analyzer version 1.27 and the hierarchy file version 1.06 [20]. A signal-to-noise ratio (S/N ratio) above two was taken as a cutoff for a positive signal. To compare values from different hybridizations, signals were normalized using the internal control DunGS02_25_dT (corresponds to *Dunaliella tertiolecta*), and replicates averaged. The mean of the total signal intensity and its standard deviation (SD) for the replicates of each probe, which are depicted in the graphs below, can be found in supplementary S2. All microarray results were uploaded to the MIDTAL database at http://www.mba.ac.uk/midtal. Specific instructions can be found in the MIDTAL manual [14] to open a new account from this site.

## 3. Results and Discussion

### 3.1. Species Composition during Sampling Period Based on Cell Counts

The samples were characterized by a mixed assembly of species (Table S1) and dominated mainly by diatoms and cryptomonads (Table 3). Dominant taxa in the five samples were *Chaetoceros* spp., *Cryptomonadales*, *Asterionellopsis glacialis* and *Cylindrotheca closterium*. The last sample (6A) by the end of October showed also a bloom of *Nitzschia* spp. With respect to potentially toxic algae (Table 4, Table S1), it was possible to observe several developments of *Pseudo-nitzschia* species. Based on morphological characteristics of the valves and on previous distinctions made by other authors [21,22], species of *Pseudo-nitzschia* were grouped and counted using four identification groups: the “slender” (*seriata* complex, *i.e.*, *P. multiseries* + *pungens*), the “thin” (valve < 3 µm, *delicatissima* complex, *i.e.*, *P. calliantha* + *delicatissima* + *pseudodelicatissima*), the “wide” (valve > 3 µm, *seriata* complex, *i.e.*, *P. australis* + *fraudulenta* + *seriata* + *subpacifica*), and the “sigmoid” (*P. multistriata*). The sigmoid group was observed with low abundances in August (sample 3A, 400 cells·L^−1^) and higher abundances in October (samples 4A and 6A) with a maximum of 40,600 cells·L^−1^ in the last sample. In July (sample 1A), it was possible to observe a high concentration of the “wide” *Pseudo-nitzschia* (*P. australis*, *fraudulenta*, *seriata*, and *subpacifica*) with 30,200 cells·L^−1^, as well as 2 cells of *Alexandrium* spp.. At the beginning of August (sample 2A) and the beginning of October (sample 4A), species of *Prorocentrum* (P. cf. *minimum*, *balticum*, and *cordatum*) were detected with 400 cells·L^−1^. Furthermore, 7,000 cells·L^−1^ of *Pseudo-nitzschia* (mainly from the “thin” group) were observed in sample 2A. Except for the aforementioned *Pseudo-nitzschia multistriata* (400 cells·L^−1^), no potentially toxic species were observed at the end of August (sample 3A). In both October samples (4A and 6A), it was possible to identify *Heterosigma akashiwo* with 600 and 400 cells·L^−1^, respectively. In the late October sample (6A), 30 cells·L^−1^ of *Dinophysis caudata* were also counted.

Despite few events of potentially toxic algae blooms during our study period, we can point out the presence of five genera in our samples (*Pseudo-nitzschia*, *Heterosigma*, *Prorocentrum*, *Alexandrium*, and *Dinophysis*) that are all represented by probes on the MIDTAL microarray.

**Table 3:** Non-toxic cells in high abundance at the Arcachon site over the sampling period in cells·L^−1^.

Species	1A	2A	3A	4A	6A
*Cryptomonadales*	50.700	331.500	181.400	194.100	36.400
*Chaetoceros spp.*	59.000	1.841.200	629.100	4.400	40.600
*Asterionellopsis glacialis*	19.000	32.400	0	27.800	446.400
*Nitzschia spp.*	1.200	400	600	11.000	73.200

**Table 4:** Cell counts of potentially harmful cells at the Arcachon site over the sampling period.

Species	1A	2A	3A	4A	6A
*Pseudo-nitzschia spp.*	30,200	7,000	0	0	800
*Pseudo-nitzschia multistriata*	0	0	400	1,700	40,600
*Prorocentrum minimum, balticum, cordatum*	0	400	0	400	0
*Heterosigma akashiwo*	0	0	0	600	400
*Alexandrium spp.*	20	0	0	0	0
*Dinophysis caudata*	0	0	0	0	30

### 3.2. Relations between Microarray Signal, Cell Counts and Detection of Toxins

The insertion of a taxonomic hierarchy file in the GPR-Analyzer [20] gave us the advantage to distinguish false positives among the species-specific probes in the microarray analysis and exclude them prior to data interpretation. Briefly, for a species to be present, the entire taxonomic hierarchy leading to that species must also be present. The slopes of culture calibration curves of each species incorporated into the GPR-Analyzer allow for the transformation of microarray signals into cell abundances.

#### 3.2.1. *Pseudo-nitzschia* and ASP Toxins

**Figure 3:**
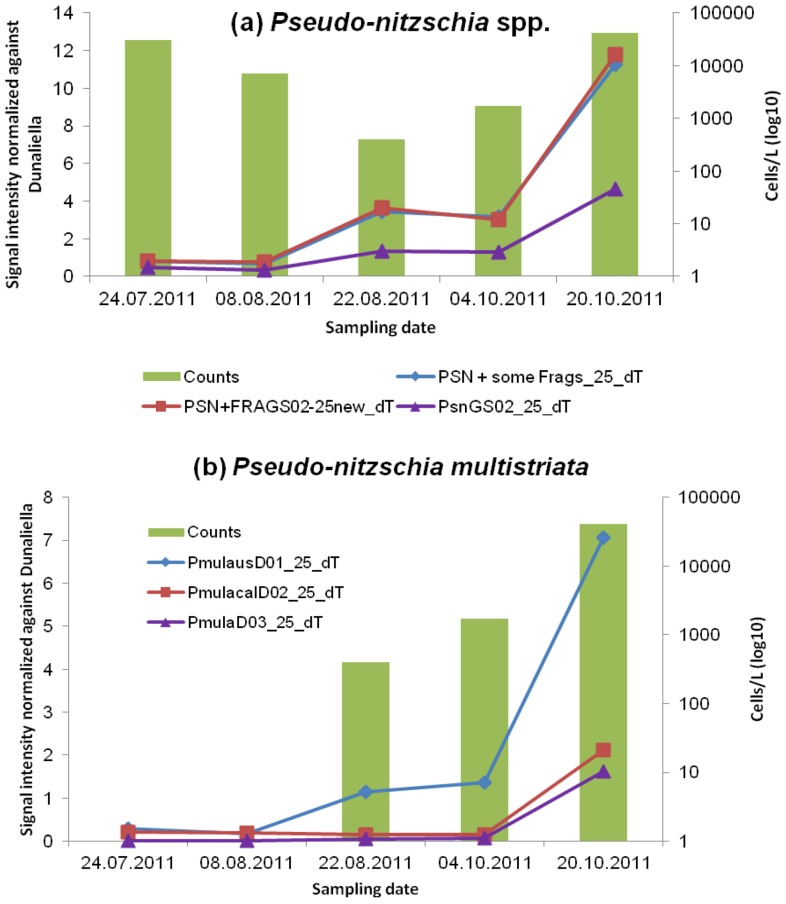
Microarray signals of (**a**) the *Pseudo-nitzschia* spp. Genus-level probes (PSN + some Frags_25_dT, PSN + FRAGS02-new_dT and PsnGS02_25_dT) and (**b**) *P. multistriata* species-level probes (PmulausD01_25_dT, PmulacalD02_25_dT, PmulaD03_25_dT) normalized against *Dunaliella tertiolecta* (DunGS02_25_dT) for the field samples taken in Arcachon Bay, France and compared to cell counts. The graphs show only probes that yielded a signal above the detection limit (signal/noise ratio > 2), except for PmulaD03_25_dT, which is only in sample 6A above the S/N ratio. The sampling dates (24.07.2011, 08.08.2011, 22.08.2011, 04.10.201 and 20.10.2011) correspond to the sampling names: 1A, 2A, 3A, 4A and 6A. Cell counts are depicted in log10 on the secondary y-axis and as columns.

*Pseudo-nitzschia* was observed throughout the sampling period and is the only potentially toxic phytoplankton genus that formed a dominant bloom according to the cell counts. The microarray detected three of five *Pseudo-nitzschia* genus-level probes (PSN + some Frags_25_dT, PSN + FRAGS02-25new_dT and PsnGS02_25_dT) throughout the sampling period (Figure 3(a)). The other two generic-level probes (PsnGS01_25_dT, and PSN no pungens_25_dT) were excluded because the S/N ratio was not always above two. These two are not as strong as the other three probes, which are positioned at the top of the hierarchy file and thus do not cause the hierarchy test to fail. Weaker probes are always placed inside stronger probes to prevent such failure of true positives. Domoic acid (DA) was detected with ELISA [23] (Table 5) in both October samples (4A and 6A) but not in sample 3A (22.08.2011) where 400 cells·L^−1^ of *P. multistriata* were counted. This result suggests that the threshold for detecting DA with ELISA is somewhere between 400 and 1,700 cells·L^−1^ for the species *P. multistriata*. Furthermore, the Multi SPR gave no signal even though the last October sample had 40,600 cells·L^−1^. In general, it was found that the ELISA was more sensitive to lower amounts of toxin than the Multi SPR [23]. Because it is quite arduous to identify *Pseudo-nitzschia multistriata* to the species-level with light microscopy, and because some of our species-specific probes are still being optimized, we focused our comparison on *P. multistriata* (*i.e*., the “sigmoid” group) with two genus-level probes and three species-level probes on the array. The October bloom of 40,600 cells·L^−1^ of *Pseudo-nitzschia multistriata* matched the microarray with positive hits (S/N ratio above 2) of the two genus-level probes (PSN + some Frags_25_dT and PSN+FRAGS02-25new_dT) and the three species-level probes (PmulausD01_25_dT, PmulacalD02_25_dT, and PmulaD03_25_dT) (Figure 3(b)). The probe PcalfrauD04_25_dT (now interpreted to be a genus-level probe because it cross-reacted with all *Pseudo-nitzschia* spp. tested) showed consistent high signals for all *Pseudo-nitzschia* spp. in calibration curves (data not shown) and field samples.

**Table 5:** Toxins measured by Multi SPR and ELISA during the sampling period in Arcachon Bay, France, adapted from [23].

	STX		Okadaic Acid, DTXS		Domoic Acid	
	(PSP)		(DSP)		(ASP)	
Sampling Date	Multi SPR	ELISA	Multi SPR	ELISA	Multi SPR	ELISA
24.07.2011	−	−	−	−	−	−
08.08.2011	−	−	−	+	−	−
22.08.2011	−	+	−	+	−	−
04.10.2011	−	+	−	+	−	+
20.10.2011	+	+	−	+	−	+

#### 3.2.2. *Dinophysis* and *Prorocentrum* and DSP Toxins

The non-toxic species *Dinophysis tripos* was counted in sample 1A (20 cells·L^−1^) and the toxic species *D. caudata* in sample 6A (30 cells·L^−1^, Table 4). No other *Dinophysis* species was identified by using light microscopy. Only the top genus-level probe in the hierarchy for *Dinophysis* (DphyGS03_25_dT) was detected with the microarray in sample 6A, but no species-specific probes were detected with the microarray in sample 6A. This result suggests that the microarray threshold for *D. caudata* species probe is above 30 cells. 

**Figure 4:**
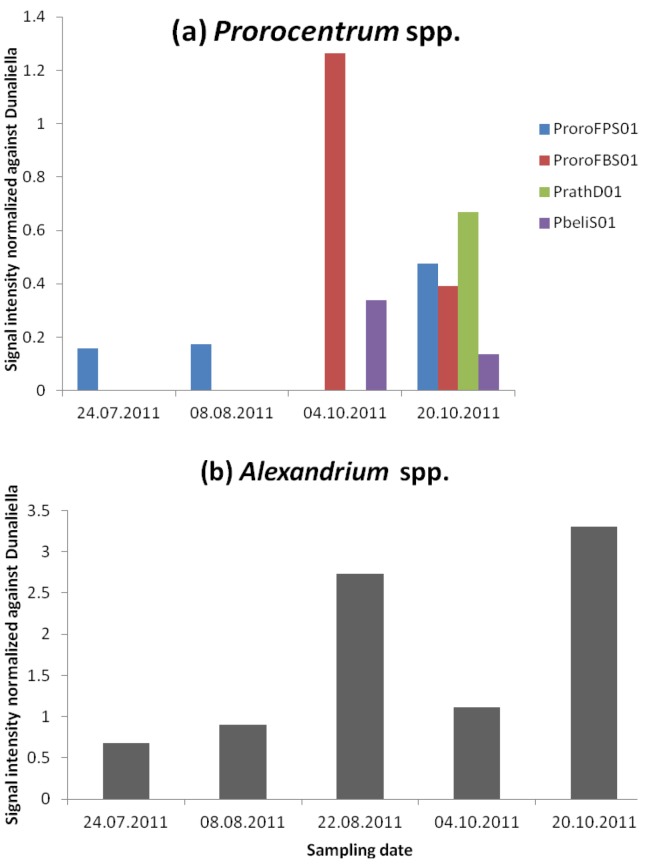
(**a**) Normalized signal of *Prorocentrum*-level probes (ProroFPS01 and ProroFBS01) and the species-level probes PrathD01 and PbeliS01. (**b**) Normalized signal of the *Alexandrium* genus-level probe AlexGD01_25_dT.

Cells from the potentially toxic genus *Prorocentrum* (group of *P. minimum*, *balticum*, and *cordatum*) were counted in samples 2A and 4A (both with 400 cells·L^−1^, Table 4). In addition, two planktonic usually considered harmless species, *P. micans* (sample 2A) and *P. triestinum* (sample 3A, 4A and 6A), were also identified by light microscopy with abundances ≤800 cells·L^−1^ (Table S1). No *Prorocentrum* species were counted in sample 1A. None of the planktonic clade-level probe for *Prorocentrum* ProroFBS02_25_dT and the species-specific probes for *P. minimum* (PminiD01_25_dT) and *P. micans* (PmicaD02_25_dT) of the microarray detected the presence of these taxa. It is likely that they require higher cell numbers to achieve a signal. With the third generation of the MIDTAL microarray new probes for *Prorocentrum* (two clade-level and six species-level probes) were tested, but without the poly dT_15 spacer region to raise the probes higher above the surface because they were still under testing for specificity. The new planktonic *Prorocentrum* probe ProroFPS01 was detected in samples 1A, 2A and 6A whereas the benthic *Prorocentrum* probe ProroFBS01 was detected in samples 4A and 6A (Figure 4(a)). New species-specific probes were made for the benthic species *P. belizeanum*, *maculosum*, *rathymum* and *mexicanum*. The probe PbeliS01 specific for *P. belizeanum* was detected with the microarray in samples 4A and 6A and the probe PrathD01 specific for *P. rathymum* and *mexicanum* was detected in sample 6A (Figure 4(a)). As for both samples, the higher probe ProroFBS01 (benthic *Prorocentrum*) was detected; the species-specific probes are not false positives and point out the limitation of microscopic cell counting. The specificity of theses *Prorocentrum* species has only been tested against a limited number of species and it is also likely that these probes are cross-reacting to another species present in the sample. *P. rathymum* is found in Malaysia and in the Mediterranean and *P. mexicanum* has a Caribbean distribution. More work is needed to clarify the taxon that is reacting with this probe. One way to achieve this is to use the probe as a FISH probe and sort the labeled cells or look at them in the microscope.

Okadaic acid was detected by ELISA in all samples except for the first (sample 1A), and the Multi SPR gave no signal at all. We presume that because *Prorocentrum* was more abundant than *Dinophysis*; its species is the source of this toxin. 

#### 3.2.3. *Alexandrium* and PSP Toxins

Two cells of the genus *Alexandrium* (*ca*. 20 cells·L^−1^) were counted only in sample 1A, whereas the microarray detected it throughout the sampling period (Figure 4(b)) with the highest signal at the end of October (sample 6A). Furthermore, PSP toxins were detected with ELISA in late August (sample 3A) and the remaining sampling period, as well as with the Multi SPR in sample 6A (Table 5, see [23] for more discussion on toxin found in these samples). If toxin probes are efficient and therefore PSP toxins are indeed present, there are two different ways to explain the absence of *Alexandrium* in cell counts: either *Alexandrium* cells have effectively been missed with the microscope, or there are other PSP-containing microorganisms in the water that are not identified. Neither *A. ostenfeldii*, *A. minutum* nor *A. tamarense* probes were detected by the microarray and their calibration curves for each specific probe have a detection limit of 200 cells [24]. Thus, we are unsure as to which species could be contributing to the PSP toxin profile. It could be *Gymnodinium catenatum* (see below) or another member of the genus *Alexandrium*. *A. pseudogonyaulax* could be a potentially missed *Alexandrium* species. There are no *A. pseudogonyaulax*-specific probes on the microarray. There are also many species that are not well investigated for toxin production. However, our data underlines the importance of including additional genus- and species-level probes for *Alexandrium*, in order to capture the full variability found in this genus. In any case, the detection of *Alexandrium* and its PSP toxins shows the advantage of the combination of the two methods (species and toxins) to detect harmful species, as well as to detect new invasive species as climate changes and tropical species move into temperate regions.

#### 3.2.4. *Heterosigma akashiwo*

The heterokont *Heterosigma akashiwo* was identified by microscopic cell counts in sample 4A (600 cells·L^−1^) and 6A (400 cells·L^−1^). The microarray detected this taxon with the species-specific probe LSHaka054425b_dT in all samples except 3A. In addition, two more species-specific probes gave positive signals in sample 4A (LSHaka0268A25_dT and LSHaka0358A24_dT), and four in sample 6A (LSHaka0268A25_dT, LSHaka0544A25c_dT, LSHaka0329A25_dT, and SSHaka0200A25_dT). This species can be difficult to identify, especially once preserved in Lugol’s. Two species-specific probes were designed from the 18S region (SSHaka) and six more from the 28S region (LSHaka) for *H. akashiwo* (Table 2, [25]). Their calibration curves show the sensitivity of each probe and point out a low affinity with the *H. akashiwo* RNA. Some probes showed no sensitivity below 5 or even 25 ng of RNA, *i.e.*, more than 700 cells are required to get a S/N ratio above two. This means that, in our case, we had around 230 cells·L^−1^ of *H. akashiwo* because not all probes were detected.

#### 3.2.5. Species Unfound by Cell Counts but Identified with Microarray and Hierarchy File

##### Fish Killing Species

**Figure 5:**
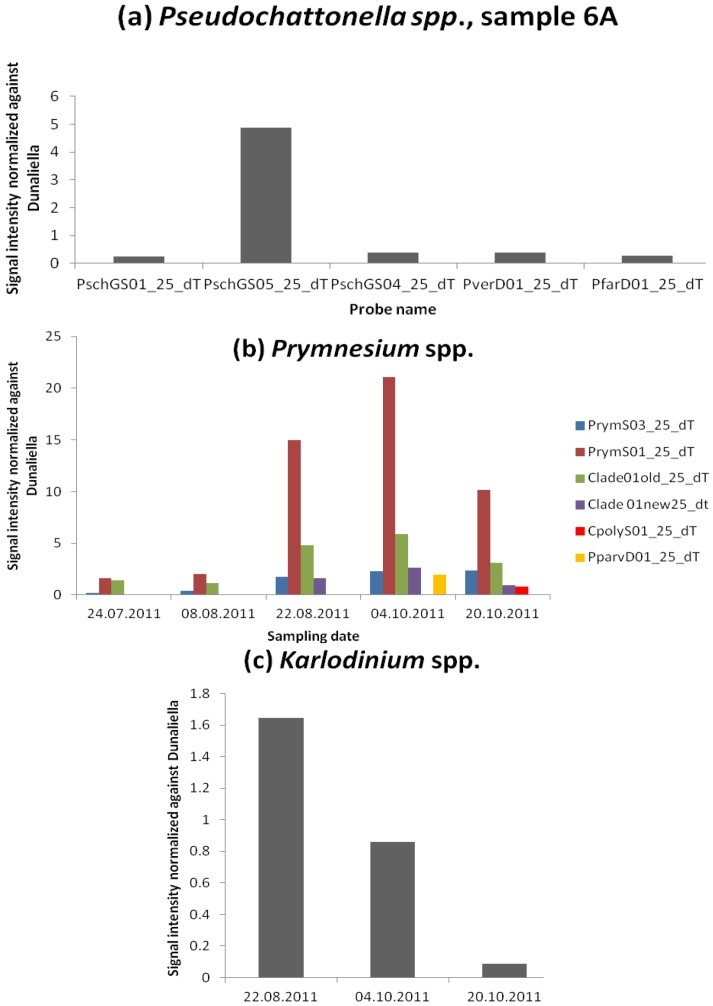
(**a**) Normalized signal intensity of the genus-level probes (PschGS01_25_dT, PschGS04_25_dT, PschGS05_25_dT) of *Pseudochattonella* and the two species-level probes PfarD01_25_dT (*Pseudochattonella farcimen*) and PverD01_25_dT (*Pseudochattonella verruculosa*) for sample 6A (20.10.2011) only. (**b**) Normalized signal intensity of the class-level probes (PrymS03_25_dT, PrymS01_25_dT) and the clade-level probe (Clade01old_25_dT) of *Prymnesium* spp. (**c**) Normalized signal intensity of the genus-level probe of *Karlodinium* spp. (KargeD01_25_Dt).

Lugol’s-fixed cells of *Pseudochattonella* are difficult to identify by light microscopy because the cell shape changes and the discharge of mucocysts gives them a warty appearance [26]. It is possible to distinguish the two sister species *P. farcimen* and *P. verruculosa* molecularly, because the two differ in several bases in the large ribosomal subunit [26]. In sample 6A, all genus-level probes of *Pseudochattonella* (PschGS01_25_dT, PschGS04_25_dT, PschGS05_25_dT) and the two species-level probes PfarD01_25_dT (*P. farcimen*) and PverD01_25_dT (*P. verruculosa*) were detected with a signal-to-noise above 2 (Figure 5(a)). The integrated calculation of cells L^−1^ in the GPR-Analyzer [20] revealed for *Pseudochattonella farcimen* 19,463 cells·L^−1^ and for *Pseudochattonella verruculosa* 48,428 cells·L^−1^, which is likely to be overestimated. Indeed, this species has only been identified in fjords and open waters of the North Sea, Skagerrak, and Kattegat, whose temperatures are below 10 °C [27]. During the summer–fall season, waters in Arcachon Bay are typically >25 °C [28]. Our results suggest perhaps another very closely related species as yet undetected could be in Arcachon Bay if the distribution of this species is exclusively in cold temperate waters. If this probe continues to show positive results, the probe could be used as a FISH probe to retrieve the cells giving the signal on the microarray for further investigations. Cells hybridized by the probe could be sorted by flow cytometry and investigated morphologically or molecularly. Once identified, the cells could later be brought into culture and their toxicity tested with bioassays.

No Prymnesiophyta were identified by cell counts, but the higher group probe for Prymnesiophyta (PrymS01_25_dT), the class level for Prymnesiophyceae (PrymS03_25_dT) and the clade-level probe for *Prymnesium* clade B1 (Clade01old_25_dT) were detected throughout the sampling period (Figure 5(b)). The second clade-level probe for *Prymnesium* clade B1 (Clade01new25_dT) was detected in samples 3A, 4A and 6A. Furthermore, the species-level probe for *P. polylepis* (CpolyS01_25_dT) was detected in sample 6A and in sample 4A the species-level probe for *P. parvum* (PparvD01_25_dT). This indicates the potential for a fish-killing event in Arcachon Bay under the appropriate conditions for growth. Although the Prymnesiophyta group is taken into account within the harmful phytoplankton monitoring program, the small size of this genus (<10 µm) as well as the smaller volume of water used for Utermöhl sedimentation and observation (100 mL maximum) than the volume of filtered seawater for RNA extraction, avoid any faithful microscopy identification and counting. The microarray can detect *Prymnesium* above 5 ng, which is equivalent to 3,800 cells for *P. polylepis* and 8,800 cells for *P. parvum* [29]. In our case (3 L filtered) it means 1,100 and 2,500 cells·L^−1^, respectively, which are high enough to be counted in a 10- or 100-mL sedimented subsample.

The genus *Karlodinium* (KargeD01_25_dT) was first detected in sample 3A and then with decreasing signals onwards (Figure 5(c)). *Karlodinium veneficum* is a high-biomass producer and the collapse of a bloom leads to the production of a surface scum that is visible as an oily, brownish discoloration of the water and kills fish and other gill-breathing animals [30]. However, no signals were detected for the six species-specific probes of *K. veneficum* present on the microarray. Based on their calibration curves (data not shown), the detection limit for four of the six probes is around 247 cells. The species-specific level probes are more sensitive than the genus-level probe. Therefore, we can exclude this species as a potential candidate being present in the bay. This is another example of how the microarray can detect potentially toxic species that are not counted or identified as being potentially toxic.

##### Azaspiracid Shellfish Poisoning (AZP) Toxins Producer

*Azadinium* spp. (AzaGS01_25_dT) was detected in sample 4A but only in two out of five spots on two different microarray slides. This may not be a genuine signal, but this species has only recently described [31] and it is also a relatively arduous species to identify based on light microscopy. Not all monitoring agencies are able to adjust their cell counts routinely to account for this toxic species. At least three more toxic species have been recently isolated and described [32,33,34].

##### Other PSP Toxins

One species-level probe out of four for *Gymnodinium catenatum* (LSGcat0270A24_dT) was detected in samples 1A, 4A and 6A. In samples 4A and 6A, the microarray also detected another species-level probe for *G. catenatum* (SSGcat0826A27_dT). The signals were not very high (S/N ratio between 2.2 and 4.9). *G. catenatum* is known to cause PSP and could therefore contribute, besides *Alexandrium*, to its detection via the ELISA and Multi SPR.

## 4. Conclusions

The third generation of the MIDTAL microarray with its improved protocols has great potential to be used as a monitoring tool for toxic algae, even in non-bloom situations, although improvements and tests are still needed. The probes on the MIDTAL microarray have been designed from a global database and the specificity tests done on the probes were made from global isolates. Thus, the MIDTAL microarray can be regarded as a universal microarray that can be used globally. Its specificity has been tested at eight sites across Europe within the MIDTAL project over a two-year period and in no case did it fail to detect the presence of a toxic species when cross-validated with the toxin array. Our results show the advantage of combining the MIDTAL microarray with toxin detection, especially for detecting species either not counted in the cell counts because of low volume or poor preservation, or because they are new to the area, *i.e.*, invasive species, such as new toxin-producing species (*Azadinium* and the causative species producing the signal for PSP) that might be new to the area or not yet routinely counted in any monitoring program. We also found several species with the microarray that were difficult to identify using light microscopy, such as *Prymnesium parvum*, *Pseudochattonella*, and *Azadinium*. A more specific identification requires electron microscopy. In other cases, such as the recording of *Karlodinium*, it is likely that the volume difference between the species filtered and settled for counting reflects the potential of the microarray to be more sensitive for the detection of rare events. 

## Appendix

**Table S1:** Cell densities (cell·L^−1^)of field samples taken at Arcachon Bay in France between July and October 2011 (1A = 26.07, 2A = 09.08, 3A = 23.08; 4A = 06.10; 6A = 20.10) and identified by light microscopy. The identified genera and species are ordered into higher taxon groups. Toxic species are indicated with an *****.

Dinoflagellates	1A	2A	3A	4A	6A
******* *Alexandrium*	20	0	0	0	0
******* *Dinophysis caudata*	0	0	0	0	30
*Dinophysis tripos*	20	0	0	0	0
*Gymnodiniaceae*	0	400		3,800	1,800
*Katodinium*	100	0	0	0	0
*Gyrodinium*	0	100	100	200	200
*Gyrodinium spirale*	0	200	0	100	0
*Prorocentrum micans*	0	100	0	0	0
******* *Prorocentrum minimum + balticum + cordatum*	0	400	0	400	0
*Prorocentrum triestinum*	0	0	200	800	400
*Protoperidinium*	0	200	0	800	600
*Protoperidinium bipes*	0	400	5,400	600	0
*Protoperidinium steinii + pyriforme*	0	0	100	0	0
*Protoperidinium diabolus*	0	0	200	0	0
*Heterocapsa niei*	0	100	1000	0	0
*Gonyaulax*	0	0	100	0	0
*Scrippsiella + Ensiculifera + Pentapharsodinium + Bysmatrum*	0	200	2,600	1,000	1,800
*Torodinium*	0	0	100	100	200
*Amphidinium*	0	0	0	200	0
*Heterocapsa triquetra*	0	0	200	0	0
*Peridiniales*	400	0	0	1,200	1,000
*Peridiniaceae*	0	0	1,400	0	0
*Peridinium quinquecorne*	0	400	3,200	0	0
**Euglena**	**1A**	**2A**	**3A**	**4A**	**6A**
*Euglenaceae*	0	600	400	0	0
*Eutreptiaceae*	0	0	0	2,200	0
*Eutreptiella*	2,400	5,000	4,000	0	3,600
**Cryptomonads**	**1A**	**2A**	**3A**	**4A**	**6A**
*Cryptomonadales*	50,700	331,500	181,400	194,100	36,400
**Diatoms**	**1A**	**2A**	**3A**	**4A**	**6A**
*Centrales*	0	0	0	0	400
*Rhizosolenia imbricata + styliformis*	0	100	0	0	0
*Rhizosolenia setigera + pungens*	0	0	0	600	1000
*Proboscia alata*	0	100	0	0	0
*Corethron*	0	0	0	100	0
*Paralia sulcata*	600	0	0	400	0
*Thalassiosira*	0	0	0	2,400	7,000
*Thalassiosira rotula*	0	0	0	0	400
*Corethron*	0	0	0	0	1,200
*Chaetoceros*	56,200	1,821,400	627,900	0	13,400
*Chaetoceros decipiens*	2,800	19,800	1,200	3,200	6,800
*Chaetoceros curvisetus + debilis + pseudocurvisetus*	0	0	0	1200	19,800
*Chaetoceros danicus*	0	0	0	0	600
*Lithodesmium*	200	5,300	7,000	3,600	6,800
*Leptocylindrus danicus*	6,200	1,600	0	3,800	12,200
*Leptocylindrus minimus*	0	4,800	0	0	6200
*Cerataulina pelagica*	400	1,000		5,400	2,800
*Dactyliosolen fragilissimus*	1,000	4,600	1200	1,000	4,400
*Guinardia striata*	1,100	0	0	0	1,800
*Guinardia flaccida*	100	0	0	0	0
*Guinardia delicatula*	0	0	0	0	1,400
*Skeletonema costatum*	0	3,600	500	0	1,000
*Odontella regia*	0	0	0	0	600
*Biddulphia alternans*	0	0	0	0	600
*Eucampia zodiacus*	0	0	0	0	1,400
*Hemiaulus*	400	1,100	0	0	0
*Lauderia*	400	0	0	0	0
*Pennales*	3,800	600	200	2,000	4,200
******* *Pseudo-nitzschia large width, seriata complex (australis + fraudulenta + seriata + subpacifica)*	30,200	200	0	0	0
******* *Pseudo-nitzschia narrow width, delicatissima complex (calliantha + delicatissima + pseudodelicatissima)*	0	6,800	0	0	0
******* *Pseudo-nitzschi, slender group, seriata complex (multiseries + pungens)*	0	0	0	0	800
******* *Pseudo-nitzschia sigmoid group (multistriata)*	0	0	400	1,700	40,600
*Thalassionema nitzschioides*	0	2,200	0	1,200	12,600
*Pleurosigma + Gyrosigma*	0	0	100	600	600
*Nitzschia*	0	0	0	1,1000	72,800
*Nitzschia longissima*	1200	400	600	0	400
*Cylindrotheca closterium*	6,600	16,400	25,350	0	2,600
*Asterionellopsis glacialis*	19,000	32,400	0	2,7800	446,400
*Licmophora*	0	0	0	100	0
*Cocconeis*	0	0	0	400	200
**Heterokonts**	**1A**	**2A**	**3A**	**4A**	**6A**
**Heterosigma akashiwo*	0	0	0	600	400
*Dictyocha*	0	0	100	400	200

**Table S2:** Mean of the total signal intensity (TI) and its standard deviation (STDEV) of each microarray-probe used in the graphs of the publication.

	1A (24.07.2011)		2A (08.08.2011)		3A (22.08.2011)		4A (04.10.2011)		6A (20.10.2011)	
	TI	STDEV	TI	STDEV	TI	STDEV	TI	STDEV	TI	STDEV
PSN + some Frags_25_dT	571,661	283,278	462,375	261,002	376,328	136,949	328,723	64,836	1,234,899	246,612
PSN+FRAGS02-25new_dT	570,012	278,217	483,590	235,672	374,378	107,819	328,689	73,975	1,236,907	237,510
PsnGS02_25_dT	366,160	122,407	269,644	59,512	176,231	40,406	154,926	102,545	527,093	47,698
PmulacalD02_25_dT	201,918	30,724	189,923	53,609	35,294	18,226	35,985	14,234	253,037	27,753
PmulaD03_25_dT	66,635	5,760	71,895	9,951	37,380	15,172	18,590	10,183	212,033	22,236
PmulausD01_25_dT	240,310	36,151	168,879	19,588	154,874	31,009	181,673	30,966	784,727	91,653
ProroFPS01	169,269	31,916	160,601	35,392	58,362	14,735			78,983	19,111
ProroFBS01							113,540	137,560	67,434	189,17
PrathD01									108,027	8,344
PbeliS01							60,137	58,069	21,412	24,719
AlexGD01_25_dT	477,054	82,900	555,744	276,647	270,780	352,356	154,715	37,017	389,966	29,238
PschGS01_25_dT									63,769	9,284
PschGS04_25_dT									547,515	98,442
PschGS05_25_dT									47,371	12,185
PverD01_25_dT									70,207	10,052
PfarD01_25_dT									51,096	8,260
PrymS01_25_dT	1,062,712	223,123	1,208,456	400,071	1,285,690	372,848	1,592,062	501,853	1,127,413	97,605
PrymS03_25_dT	184,896	64,536	262,141	132,584	204,940	51,019	231,062	63,987	285,495	49,415
Clade01old_25_dT	935,860	146,759	707,441	135,777	487,530	194,120	521,791	172,448	353,814	29,352
Clade 01new25_dt					217,043	82,294	293,107	86,528	141,925	34,971
CpolyS01_25_dT							112,327	9,400	122,730	29,900
PparvD01_25_dT							133,275	251,500		
KargeD01_25_dT					138,096	195,135	90,368	92,796	17,867	11,278
